# The Development and Psychometric Properties of a Doctoral Student Agency Scale

**DOI:** 10.3390/bs15121715

**Published:** 2025-12-11

**Authors:** Lingmei Huang, Qianqian Ruan, Kai Wang

**Affiliations:** 1 Institute of Higher Education, Faculty of Education, Beijing Normal University, Beijing 100875, China; hlm@bnu.edu.cn; 2 Department of Educational Administration and Policy, Faculty of Education, The Chinese University of Hong Kong, Hong Kong, China; 3 Center for Teacher Education Research, Faculty of Education, Beijing Normal University, Beijing 100875, China; wangkai1991@bnu.edu.cn

**Keywords:** doctoral student agency, scale development, mixed methods, doctoral education, academic socialization

## Abstract

Doctoral student agency is increasingly regarded as a key construct in doctoral education. Yet, existing research on this topic focuses on qualitative approaches, and there remains a lack of psychometrically validated instruments, particularly in the Chinese context, where supervisory authority and institutional structures strongly shape student experiences. This study aimed to develop and validate the Doctoral Student Agency Scale (DSAS) to provide a comprehensive measure of doctoral students’ agency during the process of professional socialization. A sequential mixed-methods design was adopted. First, a conceptual model was inductively constructed from semi-structured interviews with 27 doctoral students, followed by three-level qualitative coding to generate an initial pool of items. These were refined through expert review, and 436 valid responses were subjected to exploratory and confirmatory factor analyses. The final DSAS consists of 27 items organized into 7 first-order factors, which load onto 3 second-order dimensions: self-agency, academic agency, and resource agency. Moreover, DSAS scores significantly correlated with academic ability and research role identity, two critical outcomes of doctoral student professional socialization, thus confirming the criterion validity. These findings indicate that the DSAS is a valid and reliable instrument. Theoretically, it contributes to refining the multidimensional conceptualization of doctoral agency, while practically, it provides supervisors and institutions with a diagnostic tool to design targeted interventions and foster doctoral development in context-sensitive ways.

## 1. Introduction

Agency is commonly defined as an individual’s capacity to make choices, take purposeful action, and influence the trajectory of their own development (Archer, 2003; Emirbayer & Mische, 1998). In higher education, cultivating students’ agency is not only an important goal of academic development and personal growth, but also a key mechanism for achieving broader educational aims (Marginson, 2024; Vaughn, 2020). As future knowledge producers and professionals, doctoral students are expected to exercise agency in the process of professional socialization to acquire academic competence and construct scholarly identity. Yet doctoral education, as McAlpine (2020) and Hopwood and Sutherland (2009) emphasize, is a highly complex arena in which power relations, cultural norms, knowledge structures, and institutional arrangements are deeply intertwined. These complexities become particularly visible in diverse contexts; agency enables international students to navigate linguistic, institutional, and academic differences (Xu & Montgomery, 2021; Ye & Edwards, 2017), supports minority and underrepresented groups in establishing academic footholds and identity within higher education systems dominated by mainstream cultures (Gardner, 2010; Thiry et al., 2011), and helps students sustain academic progress amid the disruptions caused by the COVID-19 pandemic, such as research interruptions and social isolation (Atkinson et al., 2022). Taken together, these insights underscore that doctoral student agency is not merely an intrinsic attribute but rather a dynamic process continuously activated and constructed within specific academic and social settings.

Existing research on doctoral student agency has predominantly drawn on qualitative approaches to examine how students navigate the challenges of doctoral education. Scholars have employed agency as a lens to understand persistence, academic identity formation, and supervisory relationships, emphasizing its temporal, relational, and context-sensitive nature (Emirbayer & Mische, 1998; Hopwood & Sutherland, 2009; McAlpine, 2020). These studies provide valuable insights into how doctoral students actively negotiate structural constraints and cultural expectations, and how agency mediates their academic and professional development (O’Meara et al., 2014; Sverdlik et al., 2018). Nevertheless, despite the growing body of scholarship, agency has often been operationalized indirectly through related constructs such as motivation, self-efficacy, and other terms in psychology and management (Bandura, 2001; Garibay, 2018; Skinner et al., 1988). As a result, the concept is frequently acknowledged but rarely measured, leaving a gap in systematic and comparative assessments of doctoral student agency.

Without reliable measures, it is difficult to examine variations in doctoral students’ agency across disciplines, institutions, or cultural contexts, and equally challenging to design interventions that effectively support doctoral learning and development. To address this shortcoming, the present study aims to develop and validate the Doctoral Student Agency Scale (DSAS). By offering a theoretically grounded and empirically tested instrument, this study seeks to advance the conceptualization of agency in doctoral education and provide practical tools for enhancing supervisory practices, institutional support, and doctoral student success.

## 2. Literature Review

### 2.1. Doctoral Student Agency in Higher Education

Agency is a multifaceted concept rooted in philosophy, sociology, psychology, and education, with each discipline offering distinct yet complementary perspectives. From philosophy, agency has been historically linked to notions of free will, intentionality, and the capacity of human beings to actively transform the world (Kant, 2024; Marx, 1845). In sociology, the longstanding debate between structure and agency has generated theories such as Bourdieu’s (1990) concept of habitus and Giddens’s (2004) structuration theory, which view agency as simultaneously enabled and constrained by social structures. Psychology has emphasized the individual’s capacity for self-regulation and intentional action. Bandura (2001), for instance, highlighted personal agency, proxy agency, and collective agency, emphasizing the purposiveness, self-reflectiveness, and relational aspects of human action. Education scholars have drawn on these traditions to frame agency as a dynamic, relational, and context-dependent process, where individuals make choices and take action within the opportunities and constraints afforded by institutions, cultures, and interpersonal relationships (Biesta & Tedder, 2007; Eteläpelto et al., 2013). Despite disciplinary differences, a common thread across perspectives is that agency reflects not simply inner motivation or willpower, but the capacity to act strategically and meaningfully in relation to one’s environment.

Within doctoral education, agency is increasingly recognized as central to how students navigate the challenges of academic socialization. Doctoral programs inherently involve highly complex, long-term, and uncertainty-laden academic tasks, which require students not only to master advanced disciplinary knowledge, research competence, and higher-order thinking skills (e.g., doctoral-level research skills and research self-efficacy) (Berman & Smyth, 2015), but also to possess key behavioral competencies such as project management and time management (Elliot, 2022). Furthermore, doctoral programs involve complex interplays of power, culture, and institutional expectations, making agency essential for persistence, academic identity formation, and scholarly development (Hopwood & Sutherland, 2009; McAlpine, 2020). In this context, existing studies have provided insightful explorations of doctoral students’ agency from various perspectives, but a unified conceptual framework has not yet emerged. Using semi-structured interviews, Hakkarainen et al. (2014) proposed the notion of knowledge-creating agency, which comprises three dimensions: personal agency (reflection on one’s own abilities, sense of efficacy, and strengths and weaknesses), distributed agency (obtaining social support and scholarly guidance within the research team), and object-related agency (integrating one’s individual research into the shared themes of the group). Similarly, Goller and Harteis (2017) proposed professional agency, highlighting doctoral students’ capacity to make intentional choices and initiate actions accordingly, as manifested in handling supervisory relationships, building peer networks, and engaging in self-management. At the relational level, Hopwood and Sutherland (2009) introduced the notion of relational agency into the field of doctoral education; similarly, Jazvac-Martek et al. (2011) proposed negotiated agency, focusing on doctoral students’ capacity to elicit support from different groups of people and to establish direct academic relationships.

Despite the differing terminology, doctoral students’ agentic practices in fact converge on three key dimensions: self-management at the level of individual psychology, proactive engagement at the level of academic tasks, and the mobilization of relationships at the level of social resources. However, existing research is largely grounded in Western educational contexts, whereas Chinese doctoral students face distinctive institutional environments (e.g., expansion-related pressures, high-stakes assessment) and cultural conditions (e.g., pronounced traditions of supervisor authority) (Quan et al., 2023; Yao et al., 2025), which makes it problematic to directly apply Western concepts to the Chinese context. Moreover, current studies tend to focus on a single dimension and lack a systematic integration of these three core dimensions. Therefore, it is particularly urgent to develop a measurement instrument that can capture the individual, task, and environmental levels and that is firmly grounded in Chinese doctoral students’ experiences.

### 2.2. Measurement of Agency in Education and Doctoral Training

Efforts to measure agency have emerged across psychology, sociology, and education, though approaches remain diverse and fragmented. In psychology, researchers have frequently operationalized agency through related constructs such as self-efficacy, locus of control, or motivation (Bandura, 2001; Ryan & Deci, 2000). These measures emphasize internal beliefs and dispositions that shape individual behavior but often neglect the contextual and relational dimensions emphasized in sociological theories (Bourdieu, 1986; Giddens, 2004).

In the field of education, teachers are typically regarded as a representative group for exploring the concept of agency. Hadar and Benish Weisman (2019) conceptualized and measured teacher agency through three dimensions: self-efficacy, proactive personality, and self-promotion focus. Pyhältö et al. (2015) defined teacher professional learning agency as teachers’ deliberate and responsible management of their own and collective actions, with key factors including motivation to learn, efficacy beliefs about learning, and intentional acts for facilitating and managing learning in the professional community. This suggests that although agency is often regarded as an abstract sociological or psychological construct, empirical research in teacher education has demonstrated that translating it into concrete psychometric indicators is entirely feasible. While studies of teacher agency have proliferated, the measurement of teacher agency remains largely indirect. Researchers often adapt or infer agency through related psychological constructs—such as self-efficacy, reflection, professional identity, and learning engagement—rather than developing scales that directly operationalize the concept of agency itself. This indirect approach reflects the conceptual complexity of agency but also reveals its operational limitations, particularly in capturing the integration of intentionality, reflection, and contextual responsiveness.

A similar challenge exists in higher education research, where agency has often been indirectly assessed through proxies such as academic motivation, persistence, or identity (Sverdlik et al., 2018). While informative, such measures fail to capture agency as an integrative construct encompassing cognition, action, and structure. Existing scales also tend to target specific groups or domains—for example, professional agency among teachers (Eteläpelto et al., 2013), relational agency in collaborative contexts (Edwards, 2005) or career agency among science, technology, engineering, and mathematics (STEM) doctoral students (O’Meara et al., 2014). These targeted instruments underscore the situated nature of agency but highlight the absence of a generalizable tool that captures the multidimensional agency of doctoral students.

Within doctoral education, quantitative measurement remains limited and context-dependent. O’Meara et al. (2014) developed a survey to examine doctoral students’ career agency perspectives and actions, Hopwood and Sutherland (2009) highlighted relational agency in supervisory and peer networks, and Hakkarainen et al. (2014) conceptualized “knowledge-creating agency” to describe doctoral students’ contributions to collective research. While valuable, these approaches tend to be domain-specific, exploratory in nature, and lack systematic psychometric validation. By contrast, research in teacher education has advanced more robust frameworks and validated scales that integrate cognitive, motivational, and structural dimensions of professional agency (Biesta et al., 2015; Toom et al., 2017). These developments demonstrate that agency can be both theoretically conceptualized and empirically measured, but doctoral education lags in this regard.

In China, the gap is even more pronounced. Doctoral students are trained in a centralized, supervisor-dominated system, where their academic trajectories are shaped by hierarchical governance, project-based training, and policy-driven priorities (Li & Xue, 2022). Yet no validated tool currently exists to examine how Chinese doctoral students enact agency within such contexts. Although existing theories and empirical studies on agency have addressed different facets of doctoral students’ agency, the current literature tends to focus on a single specific dimension and lacks an integrative framework that can simultaneously capture doctoral students’ holistic agentic functioning across their internal psychological characteristics, academic practices, and interactions with resources. This absence constrains both cross-cultural comparisons and the design of interventions that support doctoral students in highly centralized systems. Moreover, without psychometrically validated instruments, it is difficult to empirically investigate how agency relates to critical outcomes of doctoral training, such as academic ability and research role identity. Addressing these limitations, the present study seeks to construct and validate a multidimensional scale of doctoral student agency, providing both theoretical refinement and practical diagnostic value.

## 3. Methods and Results

### 3.1. Research Design

It is imperative to develop a psychometrically robust scale to measure doctoral student agency that aligns with both international theoretical insights and the specific institutional conditions of Chinese doctoral education. Drawing on established theories of agency as dynamic, situated, and relational (Emirbayer & Mische, 1998; McAlpine, 2020), and acknowledging the supervisor-centered, project-based training systems prevalent in China (Dai & Elliot, 2023), this study seeks to construct the Doctoral Student Agency Scale (DSAS).

**Figure 1:**
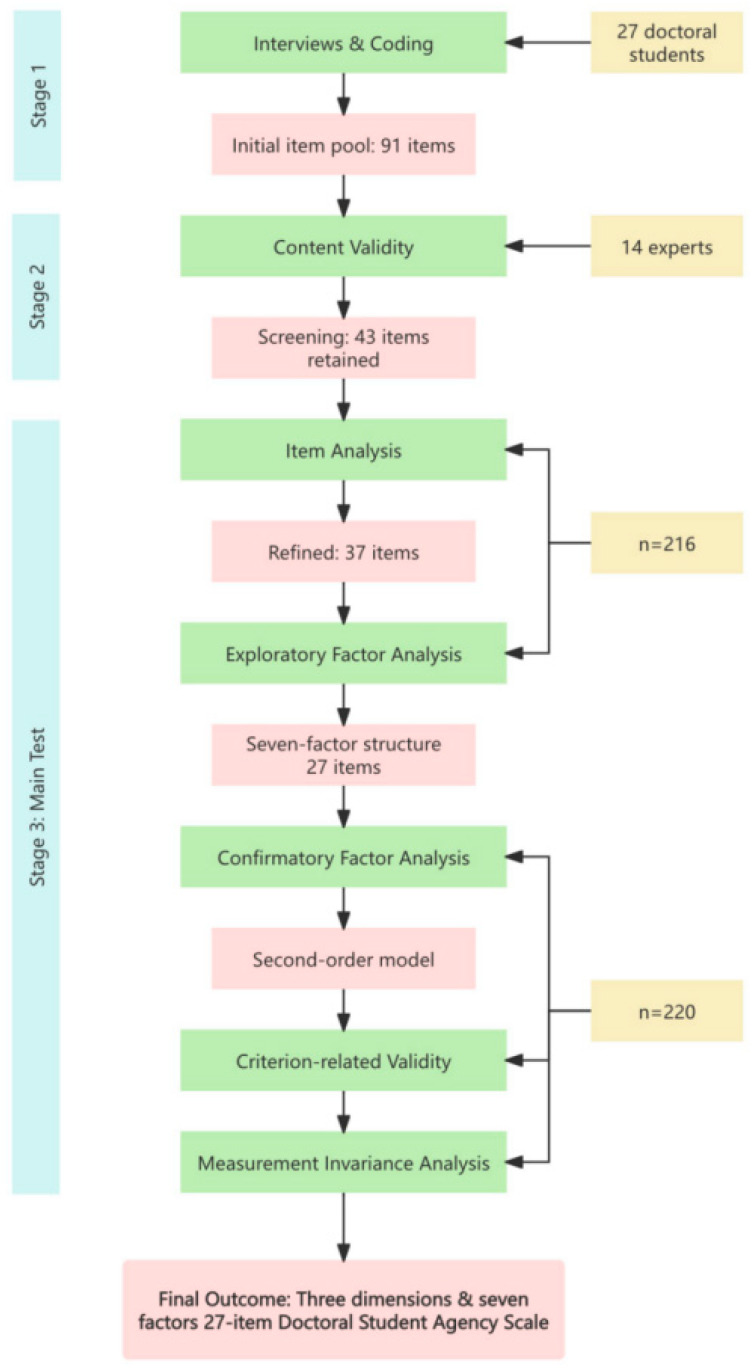
Outline of the research design.

To achieve this aim, we followed a sequential mixed methods design commonly employed in scale development research (DeVellis & Thorpe, 2021). First, semi-structured interviews with 27 doctoral students across disciplines were conducted, and a grounded theory coding procedure was applied to inductively generate a conceptual framework of doctoral agency, which served as the foundation for item generation. Second, an initial pool of items was drafted and refined through expert review to ensure content validity and contextual relevance. Third, exploratory factor analysis (EFA) was performed on Sample 1 to examine the factor structure and remove poorly performing items. Fourth, confirmatory factor analysis (CFA) was conducted on an independent dataset (Sample 2) to validate the model fit and confirm the multidimensional structure. Fifth, criterion-related validity was assessed by examining correlations between DSAS scores and theoretically relevant constructs—doctoral students’ academic ability and research role identity. Sixth, measurement invariance tests were conducted across subgroups of grade levels and disciplinary categories to evaluate the scale’s stability and cross-group applicability. Figure 1 is the outline of the research design.

Together, these procedures provide strong psychometric evidence for the DSAS as a reliable and valid instrument. The scale not only advances the conceptualization of doctoral student agency but also offers a diagnostic tool for research and practice in doctoral education.

### 3.2. Stage 1: Interviews and Coding

The structure of doctoral students’ agency was explored through interviews, which played a key role in identifying its core elements within the doctoral education context. In fact, research on the structure of doctoral students’ agency should draw on studies of agency in the field of education, including their approaches to conceptualization, factor selection, and theoretical frameworks, while also taking into account the specific context of doctoral education.

#### 3.2.1. Participants and Procedure

A total of three rounds of interviews were conducted. In the first round, open-ended interviews were carried out as a pilot to explore students’ experiences of learning, research, supervision, and life, focusing on challenges encountered and strategies of agency enactment. In the second round, the interview protocol was revised based on the themes identified in the first round, with reference to existing studies on agency and in response to issues encountered earlier, such as inappropriate questions and insufficient probing. The third round focused on disciplines that had not been covered in the previous rounds to enhance the disciplinary representativeness of the interviews.

A total of 27 doctoral students (14 female, 13 male) from three research-intensive universities in China were recruited using purposive sampling to ensure diversity in discipline (humanities, social sciences, STEM) and year of study. Details of the interview participants are provided in Appendix A. Interviews lasted 60–90 min, were audio-recorded with consent, and transcribed verbatim for coding.

To ensure the objectivity and reliability of the qualitative analysis, this study conducted an inter-rater reliability check during the open coding phase. The first author and a trained research assistant independently coded 20% of the interview transcripts, with this subsample covering different disciplines and years of study. Cohen’s kappa (κ) was subsequently used to evaluate the level of agreement between the two coders. The results showed κ = 0.86; according to the evaluation criteria proposed by Landis and Koch (1977), this value falls within the level of almost perfect agreement, indicating that the qualitative coding in this study has high reliability. After computing inter-rater agreement, the research team discussed and resolved all discrepant codes and then applied the finalized coding framework to the entire dataset.

#### 3.2.2. Results

The development of the Doctoral Student Agency Scale (DSAS) followed established scale-construction guidelines (DeVellis & Thorpe, 2021) and proceeded in three sequential phases. Semi-structured interviews were analyzed using grounded theory coding (open, axial, selective) in NVivo 12. This process yielded a three-dimensional, seven-factor framework of doctoral student agency (see Table 1): self-agency (self-cognition, self-management), academic agency (academic planning, academic autonomy), and resource agency (organizational resource utilization, interpersonal resource utilization, and student-supervisor relationship construction).

The results showed that Resource Agency (205 references) was the most frequently mentioned dimension when doctoral students discussed how they exercised agency. This category included three subdimensions: Organizational Resource Utilization (58 references), Interpersonal Resource Utilization (84 references), and Student–Supervisor Relationship Construction (63 references). Although the supervisor relationship seems to belong to interpersonal resources, participants naturally distinguished their supervisors from other social connections during interviews. Unlike other interpersonal resources (such as peers or academic networks), the supervisor relationship was characterized by high dependency and strong structural constraints; thus, it was extracted as an independent subcategory. Within this core category, seeking help actively (49 references, mentioned by 77.8% of participants) and utilizing platform resources (41 references, mentioned by 81.5% of participants) were the most widely covered behaviors. This suggests that agency was often perceived as breaking information barriers and actively reaching out for external support.

Academic Agency (176 references) emerged as another central dimension of doctoral agency, encompassing two subcategories: Academic Planning and Academic Autonomy. Academic planning was the most frequent subcategory (114 references), with strategic planning (43 references, 59.26%) and goal orientation (42 references, 66.67%) being especially prominent, indicating that doctoral students tended to advance their academic work through the establishment of clear goals and structured plans. Academic Autonomy (62 references) also played a significant role, with nearly half of the participants (40.74%) discussing the importance of academic identity formation and leading their own research.

Self-Agency comprised Self-Cognition (57 references) and Self-Management (47 references). Compared with Academic and Resource Agency, Self-Agency appeared more implicit and non-event-based, thus showing lower visibility in interview narratives. Among its categories, mental and physical well-being regulation was the most salient (39 references, mentioned by 51.85% of participants), reflecting doctoral students’ strong concern for maintaining balance under the pressure of academic life.

It should be noted that the frequency and percentage of participants mentioning codes only reflect shared perceptions and experiences within a limited sample rather than statistical significance. Higher coding frequencies represent relative consensus among participants, whereas variations may be influenced by multiple contextual factors such as academic stage, individual condition, research context, and supervisor support.

### 3.3. Stage 2: Creating Items and Establishing the Content Validity

Based on three rounds of coding of the interview data, the concept and structure of doctoral students’ agency were initially established and used as the foundation for scale development. During item design, representative statements were extracted from the interviews, while existing instruments measuring related concepts in graduate education (e.g., research self-efficacy) were consulted for reference. Item selection followed three principles: (1) alignment with the conceptual connotation of “agency”, (2) reflection of the specific characteristics of the doctoral students, and (3) consistency with participants’ own expressions whenever possible. The initial questionnaire consisted of 91 items, most of which were derived directly from the interviews, while others were carefully adapted from existing scales after an evaluation of their appropriateness. Multiple items were designed for each reference point to ensure the quality of item selection.

Content validity refers to the extent to which the items of a scale adequately represent the intended content domain (Straub et al., 2004) and is typically established through expert evaluation. To ensure content validity, experts and doctoral students were invited to review the initial item pool after its development. They were asked to evaluate each item in terms of content, wording, emotional tone, and psychometric requirements, and to provide suggestions for revision. A total of 14 participants were involved, including 6 university professors and 8 doctoral students. Based on their feedback, the initial questionnaire was revised, resulting in a 43-item Doctoral Student Agency Scale for use in the next stage of the study (see Appendix B).

### 3.4. Main Test

#### 3.4.1. Methods

##### Participants and Procedure

For the scale validation phase, participants were recruited using Wenjuanxing, the largest online survey platform in China, and distributed via WeChat, the most widely used social media platform. Participants were randomly sampled across disciplines and years of study. Inclusion criteria required that participants were current doctoral students; participation was voluntary and anonymous. Quality control measures were applied, including exclusion of responses completed in less than 5 min, patterned answers, and extreme outliers. Ethical approval for the study was obtained from the Institutional Review Board of Beijing Normal University, and all participants provided informed consent. A total of 436 doctoral students from three major comprehensive universities in China participated in this study. The samples were randomly divided into two groups for item analysis, exploratory factor analysis (n = 216) and confirmatory factor analysis, and criterion-related validity and measurement invariance (n = 220).

Disciplines represented included humanities and social sciences (69.04%), and sciences and engineering (30.96%). Among them, 157 (36%) were male and 279 (64%) were female. In terms of academic year, most doctoral students were in their first (22.71%) or fourth year (27.52%). Prior to participation, all students provided informed consent; the study protocol was approved by the institutional ethics committee, and data were collected anonymously with confidentiality assured. The demographic distribution of the sample is detailed in Table 2.

##### Data Analysis Procedure

The 43-item version was distributed online to doctoral students. Participants responded to the items using a 5-point Likert scale ranging from 1 (strongly disagree) to 5 (strongly agree). Data analyses were conducted using SPSS 26.0 and AMOS 26.0. Item analysis, EFA, reliability, and validity tests were performed in SPSS, while CFA and measurement invariance testing were conducted using AMOS. Model fit was evaluated with multiple indices: χ^2^/df, CFI, TLI, RMSEA, and SRMR, following conventional thresholds (Hu & Bentler, 1999).

The data analysis procedure consisted of four steps. (1) Item analysis and exploratory factor analysis (EFA) were conducted with the first group (n = 216). Low-quality or poorly discriminating items were removed through item analysis, while EFA was used to explore the factor structure of the DSAS and eliminate items that weakened the measurement of major factors. This stage resulted in a revised 37-item scale with seven factors. (2) Confirmatory factor analysis (CFA) was then performed with the second group (n = 220) to further validate the factor structure and assess the model fit, reliability, and validity. (3) Criterion-related validity was examined by analyzing correlations with academic ability and research role identity. (4) Measurement invariance was tested across different years of study and disciplinary groups to evaluate the scale’s stability and generalizability across subpopulations.

#### 3.4.2. Results of the Main Test

##### Item Analysis

An item analysis was conducted on the initial 43 items of the Doctoral Student Agency Scale (DSAS) to assess their psychometric quality. Three criteria were applied: (a) discrimination power (extreme group comparison), (b) item–total correlations, and (c) homogeneity tests, including Cronbach’s α if an item was deleted, as well as communalities, and factor loadings. Results indicated that six items (Items 10, 14, 20, 30, 37, and 41), most of which were reverse-worded, consistently failed to meet acceptable thresholds. Specifically, these items displayed weak discrimination indices, low item–total correlations (r < 0.40), and unsatisfactory communalities (<0.20) or factor loadings (<0.45). Deleting these items improved the internal consistency of the scale (overall α = 0.952). Consequently, six items were removed, and 37 items were retained for subsequent exploratory factor analysis (EFA). Table 3 presents the summary of item analysis results, including the criteria and deletion decisions. Based on these results, six items (10, 14, 20, 30, 37, 41) were removed, leaving 37 items for subsequent factor analysis.

##### Exploratory Factor Analysis (EFA)

Exploratory factor analysis (EFA) was conducted on the remaining 37 items using principal component analysis with varimax rotation. The results of the 7 common factors extracted through the initial EFA are presented in Appendix C. The Kaiser–Meyer–Olkin (KMO) measure of sampling adequacy was 0.931, and Bartlett’s test of sphericity was significant (χ^2^ = 5010.21, *p* < 0.001), indicating that the data were suitable for factor analysis. Factor retention was based on eigenvalues greater than 1, the scree plot, and the interpretability of factors in line with the theoretical framework.

After multiple iterations of item screening, ten items were deleted due to low factor loadings (<0.45), high cross-loadings, or weak communalities. The final solution yielded seven factors comprising 27 items, which together explained 70.06% of the total variance. After varimax rotation, the eigenvalues of the seven factors ranged from 2.18 to 3.31, and the variance explained by each rotated factor ranged from 8.061% to 12.257% (see Table 4). The seven factors were as follows: Academic Planning, Interpersonal Resource Utilization, Academic Autonomy, Student–Supervisor Relationship Construction, Self-Management, Organizational Resource Utilization, and Self-Cognition. Table 4 reports the final factor loadings.

##### Confirmatory Factor Analysis (CFA)

To further test the structural validity of the Doctoral Student Agency Scale (DSAS), confirmatory factor analyses (CFA) were conducted using AMOS 26.0 with maximum likelihood estimation. Two competing models were tested: a first-order seven-factor model based on the results of the exploratory factor analysis (EFA), and a second-order model comprising three higher-order dimensions—Self-Agency, Academic Agency, and Resource Agency—encompassing seven lower-order factors (Self-Cognition, Self-Management, Academic planning, Academic autonomy, Organizational Resource Utilization, Interpersonal Resource Utilization, and Student–Supervisor Relationship Construction).

**Figure 2:**
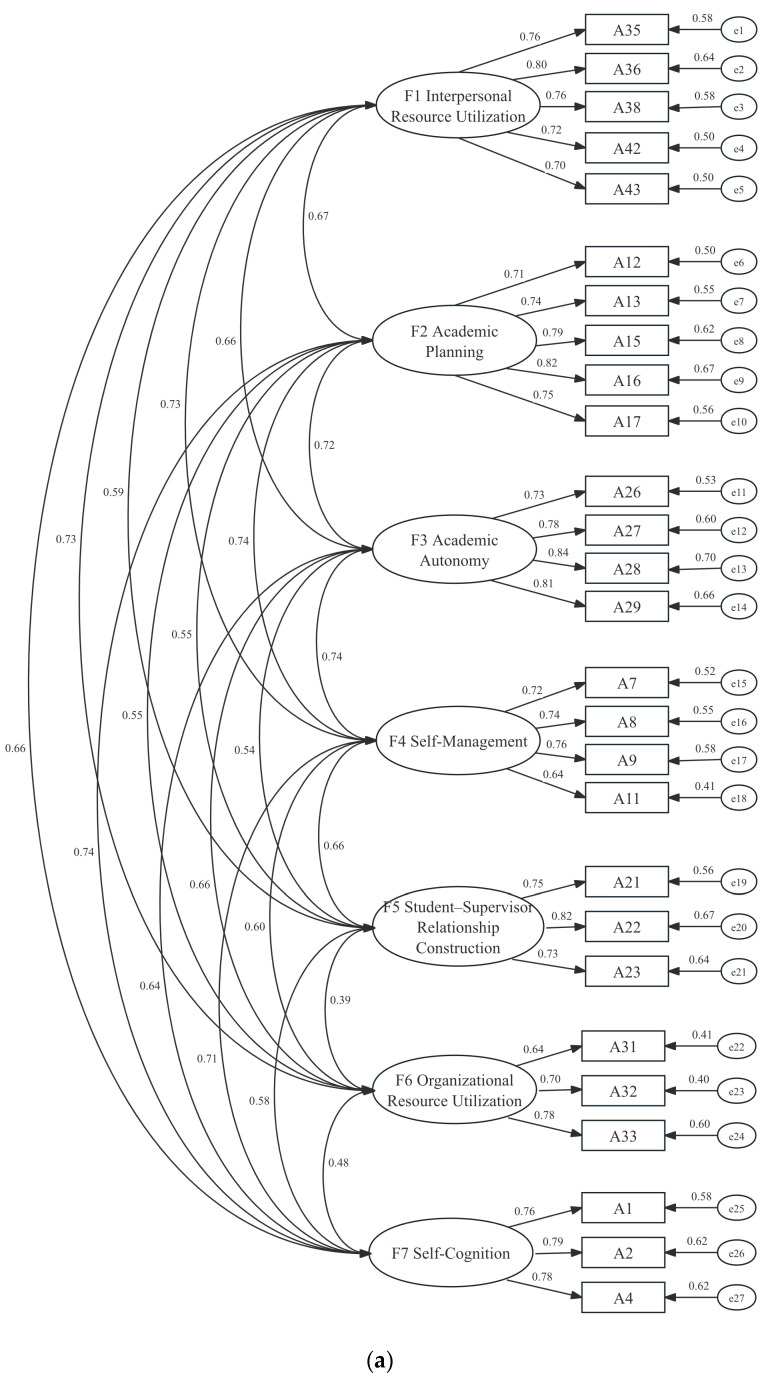

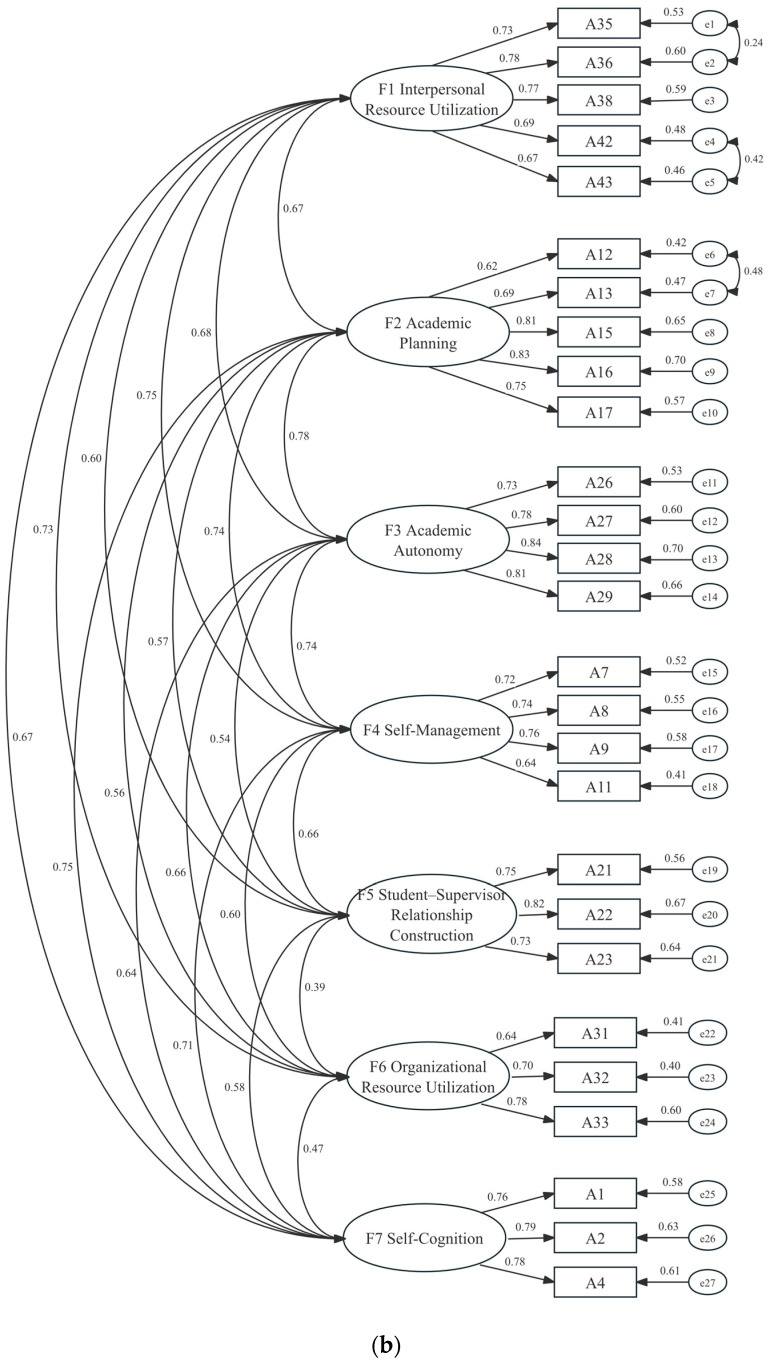
(**a**) The first-order seven-factor structure model (before revision); (**b**) the first-order seven-factor structure model (after revision).

The initial fit indices of the first-order seven-factor model (see Figure 2a) were acceptable but could be improved (χ^2^ = 809.23, df = 303, χ^2^/df = 2.671, GFI = 0.874, CFI = 0.922, TLI = 0.909, RMSEA = 0.062). Guided by modification indices and theoretical justification, error covariances were added among several residuals, which significantly enhanced the model fit. The revised first-order seven-factor model demonstrated good fit: χ^2^ = 614.026, df = 300, χ^2^/df = 2.047, GFI = 0.905, CFI = 0.951, TLI = 0.943, RMSEA = 0.049. The revised model is presented in Figure 2b.

**Figure 3:**
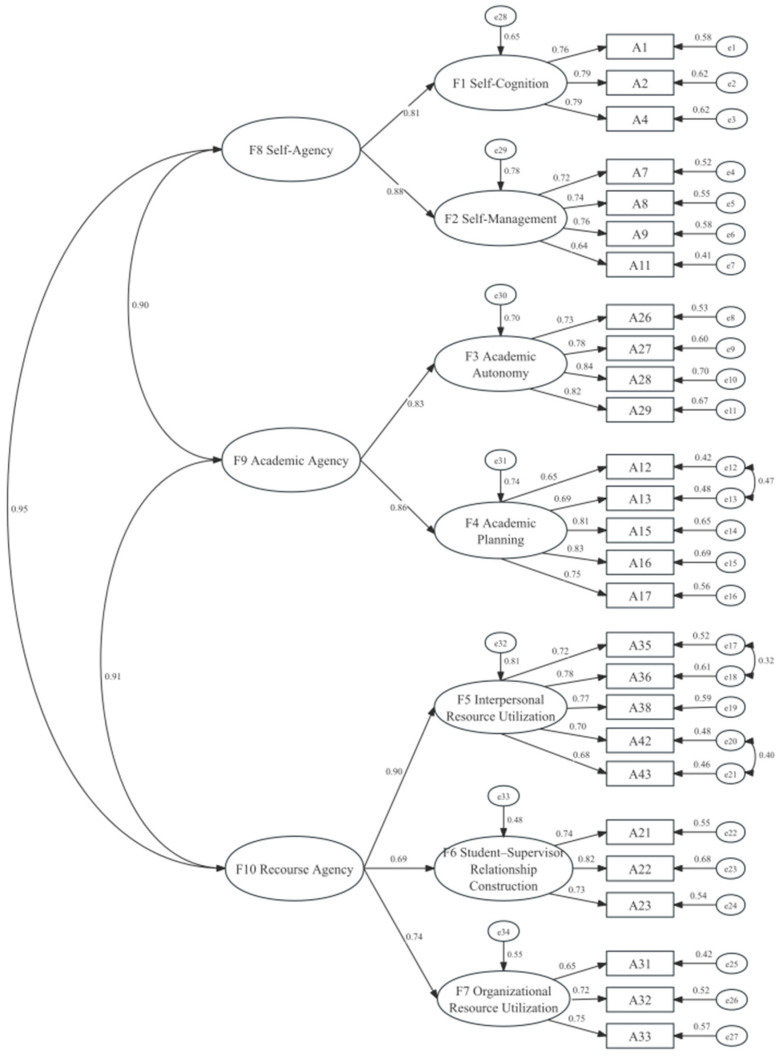
The second-order structure model (after revision).

Similarly, the second-order model was evaluated (see Figure 3). The initial fit indices indicated acceptable fit (χ^2^ = 864.316, df = 314, χ^2^/df = 2.753, GFI = 0.867, CFI = 0.915, TLI = 0.905, RMSEA = 0.063). After model modification, the revised second-order three-dimension seven-factor model showed improved fit, with all indices meeting recommended thresholds (χ^2^ = 650.773, df = 310, χ^2^/df = 2.099, GFI = 0.900, CFI = 0.947, TLI = 0.940, RMSEA = 0.050).

Both the first-order and second-order models demonstrated satisfactory model fit, but the second-order model provided a more parsimonious structure consistent with theoretical expectations. These results support the multidimensional and hierarchical nature of doctoral student agency, validating the three-dimension, seven-factor structure of the DSAS. Importantly, the decision to adopt a second-order structure was justified both theoretically and statistically. Theoretically, the qualitative coding framework revealed three overarching domains of agency—Self-Agency, Academic Agency, and Resource Agency—each encompassing multiple first-order dimensions. This hierarchical logic is consistent with prior research on professional and educational agency, which has frequently employed higher-order models to capture the integration of personal, relational, and structural elements (Eteläpelto et al., 2013; Vähäsantanen, 2015). Statistically, the inter-factor correlations among the seven first-order factors were moderate to strong (r = 0.31–0.64), suggesting the presence of higher-order latent constructs. Moreover, the target coefficient for the second-order model was 0.944, indicating that the higher-order solution accounted for nearly all the covariation among the first-order factors. Together, these findings support the adoption of a three-dimension, seven-factor second-order model as a parsimonious yet conceptually robust representation of doctoral student agency.

The internal consistency reliability of the Doctoral Student Agency Scale (DSAS) was examined using Cronbach’s α coefficients for the overall scale and its seven subscales. The Cronbach’s α for the full 27-item scale was 0.941, exceeding the recommended threshold of 0.90 and indicating excellent reliability. Across the seven subscales, α values ranged from 0.752 to 0.878, with Self-Cognition (α = 0.779), Self-Management (α = 0.799), and Organizational Resource Utilization (α = 0.752) demonstrating good reliability, while Student–Supervisor Relationship Construction (α = 0.805), Academic autonomy (α = 0.862), Academic planning (α = 0.878), and Interpersonal Resource Utilization (α = 0.858) showed very good reliability. These results provide robust evidence that the DSAS possesses satisfactory internal consistency at both the scale and subscale levels.

##### Criterion Validity

Two theoretically relevant outcomes—academic ability and research role identity—were used to assess the criterion validity of the Doctoral Student Agency Scale (DSAS). Academic ability, measured with a revised 14-item scale (Chi, 2017), captures doctoral students’ academic knowledge, skills, and practice, while research role identity, measured with a 6-item adapted scale (D. Fu, 2020; L. Fu & Charoensukmongkol, 2021; Zhang, 2014), reflects the extent to which doctoral students internalize their scholarly roles. Both instruments demonstrated satisfactory reliability (α > 0.80).

As shown in Table 5 and Table 6, DSAS total scores were strongly and positively correlated with both academic ability (r = 0.70, *p* < 0.001) and research role identity (r = 0.59, *p* < 0.001). At the subscale level, Self-Management and Self-Cognition showed the strongest associations with academic ability, while Self-Cognition and the higher-order Self-Agency dimension were most predictive of research role identity. Comparatively, DSAS exhibited stronger associations with academic ability than with research role identity, but both results highlight that agency plays a pivotal role in doctoral students’ competence and identity development. These findings provide robust evidence for the criterion-related validity of the DSAS.

##### Measurement Invariance

To evaluate whether the Doctoral Student Agency Scale (DSAS) provides meaningful comparisons across subgroups, measurement invariance tests were conducted across grade levels (early vs. advanced years) and disciplinary categories (humanities & social sciences, sciences & engineering). Establishing invariance ensures that observed group differences reflect substantive variations in agency rather than measurement bias (Vandenberg & Lance, 2000).

Following standard practice (Chen, 2007; Cheung & Rensvold, 2002), we sequentially tested configural, metric, scalar, and strict invariance models. Configural invariance examines whether the same factor structure holds across groups, metric invariance constrains factor loadings to be equal, scalar invariance further constrains intercepts, and strict invariance additionally constrains residuals. Good model fit was determined by conventional cutoffs (χ^2^/df < 3, CFI/TLI ≥ 0.90, RMSEA ≤ 0.06, SRMR ≤ 0.08). Invariance was evaluated based on incremental changes in fit indices (ΔCFI ≤ 0.01, ΔTLI ≤ 0.01, ΔRMSEA ≤ 0.015).

Grade-level comparison: As shown in Table 7, all four models demonstrated acceptable fit. The shift from configural to metric and from metric to scalar models produced negligible changes in fit indices (ΔCFI ≤ 0.01, ΔRMSEA ≤ 0.001). Moving from scalar to strict invariance slightly reduced CFI (ΔCFI = −0.008), but the changes remained within recommended thresholds. These results indicate that strict invariance holds across grade levels, confirming that doctoral students at different stages of study interpret the DSAS items equivalently.

Discipline-level comparison: Similarly, invariance was supported across disciplinary categories (see Table 8). The configural, metric, scalar, and strict models all exhibited satisfactory fit, with incremental changes in indices consistently below the recommended thresholds (ΔCFI ≤ 0.01, ΔRMSEA ≤ 0.001). Thus, the DSAS achieved strict invariance across disciplines as well.

Together, these results demonstrate that the DSAS possesses robust measurement invariance across both grade levels and disciplinary groups. This finding highlights the scale’s utility for comparative research, allowing scholars and practitioners to reliably examine variations in doctoral student agency across academic stages and fields of study. Importantly, the demonstration of strict invariance indicates that observed differences in mean scores can be interpreted as reflecting substantive group differences rather than measurement artifacts, thereby enhancing the scale’s value for both theory building and policy design.

## 4. Discussion

### 4.1. Summary of Key Findings

This study employed a sequential mixed-methods design to develop and validate the Doctoral Student Agency Scale (DSAS). To overcome the construct incompleteness that may arise from relying solely on existing literature or adapting existing instruments, we first conducted in-depth coding and refinement of qualitative interview data to derive the construct dimensions and initial items from doctoral students’ experiences. Subsequently, experts rigorously reviewed the content to ensure the theoretical appropriateness and content validity of the scale. Finally, exploratory and confirmatory factor analyses were used to confirm the robustness of the scale’s structure and its sound psychometric properties. This methodological systematicity and progression effectively integrated the depth of qualitative inquiry with the breadth of quantitative analysis, ensuring that the scale meaningfully fills a key gap in higher education research, particularly in the context of doctoral education. The results confirmed a clearly structured, second-order scale with three dimensions and seven factors. The three higher-order dimensions—Self-Agency (self-cognition, self-management), Academic Agency (academic planning, academic autonomy), and Resource Agency (organizational resource utilization, interpersonal resource utilization, student-supervisor relationship construction)—together capture the multidimensional and dynamic nature of doctoral agency. Reliability analyses demonstrated high internal consistency (α = 0.941 for the full scale), and criterion-related validity was confirmed through significant correlations with academic ability and research role identity. Importantly, measurement invariance tests indicated strict invariance across grade levels and disciplinary groups, supporting the DSAS as a tool for meaningful subgroup comparisons.

This study revealed that doctoral students’ agency is a multilayered construct shaped by the interaction between the individual, academic tasks, and the broader academic environment. The findings of this study are consistent with existing measures of agency. For example, Jääskelä et al. (2017) distinguish between individual agentic resources (including interest and motivation, self-efficacy, etc.) and relational sources of agency (teacher support, peer support), which closely aligns in substance with the constructs of self-agency and resource agency in the present study. The Mindful Agency scale developed by Wang and Peng (2017) focuses on internal regulatory strategies such as learning methods, emotion regulation, and planning awareness, which align with the internal structure of self-agency and academic agency identified in this study. Whereas previous scales typically concentrated on a single facet of agency, the DSAS systematically integrates and validates agency across three dimensions within the specific and complex context of doctoral education, thereby offering a more comprehensive and theoretically informative understanding of doctoral students’ agency.

Self-agency is underpinned by two key components: self-cognition and self-management. Previous studies have often measured self-agency together with task-related agency, as in the Agency for Learning Questionnaire, which encompasses intentionality (planning and decision-making), forethought, self-regulation, and self-efficacy (Code, 2020). The present study further differentiates these components. For doctoral students, agency begins with self-cognition and self-evaluation. From the very outset, the doctoral journey requires an ongoing cycle of self-assessment, reflective adjustment, and recalibration in order to achieve continuous learning and progressive improvement (Elliot, 2022). Individuals establish intrinsic motivation through perceived value and meaning, evaluate their own capabilities through efficacy beliefs, and determine their courses of action through an awareness of their strengths and weaknesses. Meanwhile, self-management is also crucial, encompassing the executive to translate evaluation into concrete action, to reflect on and synthesize past experiences, and to regulate one’s physical and psychological state in high-pressure environments. Given the high levels of stress and anxiety commonly reported among doctoral students (Levecque et al., 2017), self-management—particularly the regulation of physical and mental states—constitutes an essential agentic resource for sustaining an academic career in highly competitive environments marked by strong external expectations.

Academic agency consists of academic planning and academic autonomy. Academic planning aligns closely with the cyclical process of planning, performance, and reflection emphasized in the self-regulated learning (SRL) model (Zimmerman, 2002). The doctoral period is often characterized by high expectations and challenges, as well as the loneliness and protracted nature of the doctoral journey, which may exacerbate feelings of uncertainty, confusion, and even fear (Elliot, 2022). Clear and effective goals and plans, strong execution, and ongoing reflection and adjustment are critical strategic tools for doctoral students to cope with ambiguity, allocate their time and energy appropriately, and ensure the sustainable progress of their research. Recognizing academic autonomy elevates agency from a set of mere behavioral strategies to the proactive construction of a research identity. Existing studies have shown that autonomy is associated with persistence in doctoral study, satisfaction, and research self-efficacy (Van Rooij et al., 2021). By defending their scholarly positions and taking the lead in their research processes, doctoral students establish autonomy and thereby complete the transition from passive recipients of knowledge to active designers of their research careers.

Resource agency highlighted that doctoral students’ agency is also expressed through their interactions with the social and academic environment. As Bandura (2001) argued, individual, behavior, and environment operate in reciprocal interaction, and the present study similarly demonstrated that agency is not merely an internal personal attribute but is also manifested through engagement with external contexts. Doctoral students actively integrated institutional, supervisory, and peer resources to compensate for limitations in their individual agency, which is consistent with recent perspectives that conceptualize doctoral education as an ecosystem (McAlpine & Norton, 2006). Through regular and direct engagement in academic communities, doctoral students gain both academic and non-academic learning opportunities, such as the formation of academic identity, the accumulation of research experience, and access to social and psychological support (Mantai, 2017). Unlike ordinary interpersonal relationships, the proactive construction of the supervisor–doctoral student relationship is particularly crucial in doctoral education. In the Chinese higher education system, characterized by high power distance, strong supervisory authority, and an emphasis on “respecting teachers,” supervisors’ disciplinary reputation, social networks, and connections with journal editors and academic organizations directly shape students’ access to hidden opportunities (Li et al., 2025). Therefore, the active and positive cultivation of the supervisor–student relationship constitutes a core aspect of the doctoral trajectory.

In sum, doctoral students’ agency should not be viewed as a single psychological trait but as a complex system shaped by self-cognition and regulation, academic planning and autonomy, and resource integration. These findings provide not only a theoretical foundation for future studies on agency but also practical entry points for strengthening doctoral training.

### 4.2. Theoretical Implications

The DSAS makes three theoretical contributions. First, it confirms established elements of agency, such as self-regulation and autonomous learning, while extending the construct through the identification of Resource Agency as a distinct higher-order dimension. Whereas prior studies often treated supervisory relationships and institutional resources as background conditions (Cornér et al., 2023; Pyhältö et al., 2012), our results empirically demonstrate that the mobilization of organizational and interpersonal resources constitutes a direct expression of agency. Notably, the supervisor–student relationship emerged as an independent factor, rather than a subcomponent of academic autonomy. This highlights the relational mechanisms by which doctoral students enact agency, especially within hierarchical systems where supervisors serve as gatekeepers of academic, social, and career resources.

Second, this finding underscores that doctoral agency is not solely an individual capacity but a relational and contextual practice, enacted through negotiation and resource mobilization within asymmetrical power dynamics. This perspective aligns with sociocultural theories that view agency as distributed across persons and structures (Edwards, 2005; Emirbayer & Mische, 1998). The recognition of resource agency bridges individualistic and structural accounts of agency, demonstrating that students’ capacity to act emerges from the interaction between personal dispositions and institutional affordances.

Third, by situating agency in the Chinese context, the DSAS challenges universalistic assumptions that equate agency with autonomy and independence. Instead, Chinese doctoral students often enact agency by strategically leveraging resources, cultivating supervisory trust, and adapting to institutional expectations, rather than by resisting them. This culturally mediated enactment enriches global theorization of agency, suggesting that in centralized and hierarchical systems, agency is better understood as negotiated and adaptive rather than purely autonomous (Marginson, 2019; McAlpine, 2020). In this sense, the DSAS contributes to comparative and international research by demonstrating how agency manifests differently across educational and cultural contexts.

### 4.3. Practical Implications

The DSAS also provides actionable insights for doctoral training. At the student level, the scale can help individuals identify their strengths and weaknesses across different domains—whether in self-management, long-term planning, or resource mobilization—and thus serves as a practical guide for targeted self-improvement. At the supervisory level, mentors can use the DSAS diagnostically to tailor guidance, for example, by fostering self-regulation strategies, supporting academic autonomy, or strengthening trust and communication within supervisory dyads. At the institutional level, universities can deploy the DSAS for program-level assessments, monitoring patterns of agency across cohorts and identifying at-risk groups who may require targeted interventions. Policy initiatives aimed at enhancing doctoral education quality may also integrate DSAS-based diagnostics to evaluate the effectiveness of reforms. In these ways, the DSAS offers a multi-level action framework for fostering persistence, innovation, and career readiness among doctoral students (Gardner, 2009; Sverdlik et al., 2018).

## 5. Limitations and Future Directions

Despite its contributions, several limitations should be acknowledged. First, the sample (N = 436) met psychometric requirements but was limited in regional and institutional diversity. Future research should include larger and more representative samples across multiple universities, disciplines, and geographic areas. Second, while expert review was conducted, the development process did not fully employ structured consensus methods such as the Delphi technique, which may explain discrepancies between the initial qualitative framework and the final factor structure. Third, the cross-sectional design precludes longitudinal inferences. As agency is inherently dynamic, future research should adopt longitudinal or experimental designs to track developmental trajectories and examine causal mechanisms (McAlpine & Amundsen, 2009). Fourth, although measurement invariance was confirmed across subgroups within China, cross-cultural equivalence was not tested. Comparative studies should examine whether the DSAS retains its structure in other contexts, thereby enriching international theorization of agency (Jääskelä et al., 2021). Finally, only two criterion variables (academic ability and research role identity) were tested. Future research could extend criterion validation to outcomes such as psychological well-being, innovation capacity, and career adaptability, which are increasingly important in doctoral education (Sverdlik et al., 2018).

## 6. Conclusions

This study developed and validated the Doctoral Student Agency Scale (DSAS) through a sequential mixed-methods design. Beginning with qualitative interviews and grounded coding, a conceptual model of doctoral agency was constructed and subsequently refined into a psychometrically robust scale through expert review, exploratory and confirmatory factor analyses, criterion-related validity testing, and measurement invariance assessments. The final DSAS consists of 27 items organized into three higher-order dimensions—self-agency, academic agency, and resource agency—across seven first-order factors, and demonstrated high internal consistency and strong model fit. The findings advance theoretical understanding by conceptualizing doctoral agency as a multidimensional construct situated at the intersection of self-regulation, academic engagement, and contextual resource integration. Practically, the DSAS offers supervisors and institutions a diagnostic tool for assessing doctoral students’ agency and designing targeted interventions that strengthen persistence, identity formation, and professional growth. Taken together, this study provides a validated instrument and a conceptual framework that can inform both future research and doctoral education practices, particularly in contexts where institutional structures and supervisory dynamics strongly shape student experiences.

## Figures and Tables

**Table 1:** Results of doctoral students’ agency coding.

Core Category	Subcategory	Initial Category	Ref. ^1^	Ref. % ^2^	Part. ^3^	Part. % ^4^
Self-agency (104)	Self-Cognition (57)	Value Perception	20	4.12%	9	33.33%
		Efficacy Perception	16	3.30%	8	29.63%
		Strengths and Weaknesses Assessment	21	4.33%	8	29.63%
	Self-Management (47)	Reflection and Experience Summarization	8	1.65%	6	22.22%
		Mental and Physical Well-being Regulation	39	8.04%	14	51.85%
Academic Agency (176)	Academic Planning (114)	Strategic Planning	43	8.87%	16	59.26%
		Adaptive Adjustment	6	1.24%	6	22.22%
		Goal Orientation	42	8.66%	18	66.67%
		Task Coordination and Management	13	2.68%	9	33.33%
		Execution	10	2.06%	6	22.22%
	Academic Autonomy (62)	Academic Identity Formation	31	6.39%	11	40.74%
		Refining Academic Integrity	8	1.65%	7	25.93%
		Leading One’s Research	23	4.74%	11	40.74%
Resource Agency (205)	Organizational Resource Utilization (58)	Understanding External Regulations	17	3.51%	10	37.04%
		Utilizing Platform Resources	41	8.45%	22	81.48%
	Interpersonal Resource Utilization (84)	Constructing Academic Networks	34	7.01%	13	48.15%
		Engaging in Knowledge Sharing	33	6.80%	16	59.26%
		Collaborating with Others	17	3.51%	9	33.33%
	Student-Supervisor Relationship Construction (63)	Seeking Help Actively	49	10.10%	21	77.78%
		Relationship maintenance and trust-building	14	2.89%	9	33.33%

Note: ^1^ Ref. Count = number of coding references; ^2^ Ref. % = % of total coding references (*N* = 485); ^3^ Part. Count = number of participants mentioning the code; ^4^ Part. % = % of total participants (*N* = 27).

**Table 2:** Demographic characteristic (N = 436).

Variable	Category	N	%
Gender	Male	157	36%
	Female	279	64%
Subject Categories	Science & Engineering	135	30.96%
	Humanities & Social Sciences	301	69.04%
Year of Study	First Year	99	22.71%
	Second Year	71	16.28%
	Third Year	83	19.04%
	Fourth Year	120	27.52%
	Fifth Year PhD and above	63	14.46%
Admission Method ^1^	Standard Admission	371	85.09%
	Special Admission	65	14.91%
Work Experience	Research-related	78	17.89%
	Non-research-related	83	19.04%
	No work experience	275	63.07%
Subject Level ^2^	First-class discipline	337	77.29%
	Non-first-class discipline	99	22.71%

Note: ^1^ In Chinese doctoral admissions, the standard admission is the standard channel open to all eligible applicants, following a formal “application–assessment” procedure with fixed quotas, uniform requirements, and unrestricted post-graduation career choices; in contrast, special admissions target clearly defined groups (e.g., direct-entry doctoral students from the undergraduate level, designated or employer-sponsored doctoral candidates, and ethnic minority programs), use quotas that are separate from the regular intake, impose more specific or stringent entry requirements, and in some cases obligate graduates to return to their original institution or region for service. ^2^ The term “First-Class Discipline” refers to a discipline designated by the Chinese government as part of the Double First-Class Initiative, a national strategy launched in 2015 to promote the development of world-class universities and world-class disciplines. Disciplines recognized under this initiative receive prioritized policy support and funding with the goal of enhancing their global competitiveness and academic excellence.

**Table 3:** Summary of item analysis results.

Item No.	Critical Ratio (t)	Item–Total Correlation (r)	Communality	Loading	α if Deleted	Decision
10	4.63	0.36	0.10	0.32	↑	Deleted
14	6.49	0.48	0.20	0.45	↑	Deleted
20	2.23	0.24	0.04	0.19	↑	Deleted
30	6.84	0.42	0.16	0.40	↑	Deleted
37	4.12	0.33	0.07	0.27	↑	Deleted
41	3.83	0.28	0.05	0.23	↑	Deleted
Others	>5.00	0.40–0.76	0.26–0.77	0.45–0.83	—	Retained

Note. ↑: Cronbach’s α increased when the item was deleted; —: Cronbach’s α remains unchanged if the item is deleted. Thresholds applied: r ≥ 0.40, communality ≥ 0.20, factor loading ≥ 0.45. Six items (10, 14, 20, 30, 37, and 41) were removed. The final pool retained 37 items for exploratory factor analysis.

**Table 4:** Factor loadings of DSAS.

Item No.	F1	F2	F3	F4	F5	F6	F7	Communality
12	0.795							0.753
13	0.736							0.722
16	0.676							0.734
17	0.624							0.621
15	0.581							0.719
35		0.818						0.788
38		0.733						0.709
36		0.710						0.715
42		0.599						0.679
43		0.515						0.583
27			0.778					0.751
28			0.726					0.705
29			0.716					0.712
26			0.714					0.674
22				0.810				0.753
21				0.779				0.746
23				0.714				0.644
11					0.724			0.708
7					0.653			0.638
8					0.633			0.706
9					0.590			0.602
31						0.759		0.692
32						0.710		0.687
33						0.592		0.706
1							0.835	0.768
2							0.692	0.718
4							0.516	0.640
RE	3.309	3.309	3.002	2.516	2.32	2.285	2.176	
CVE (%)	12.257	12.254	11.118	9.319	8.593	8.463	8.061	

Note: F1 = Academic Planning; F2 = Interpersonal Resource Utilization; F3 = Academic Autonomy; F4 = Student–Supervisor Relationship Construction; F5 = Self-Management; F6 = Organizational Resource Utilization; F7 = Self-Cognition. RE = Rotated Eigenvalues; CVE (%) = percentage of common variance explained.

**Table 5:** Correlations between doctoral student agency and academic ability.

Dimension	Academic Foundation & Accumulation	Academic Conception & Practice	Academic Management & Expression	Mean Academic Ability
First-order				
Self-Cognition	0.397 ***	0.529 ***	0.444 ***	0.534 ***
Self-Management	0.479 ***	0.598 ***	0.545 ***	0.626 ***
Academic planning	0.428 ***	0.533 ***	0.434 ***	0.540 ***
Academic autonomy	0.487 ***	0.542 ***	0.482 ***	0.576 ***
Organizational Resource Utilization	0.457 ***	0.496 ***	0.448 ***	0.532 ***
Interpersonal Resource Utilization	0.418 ***	0.544 ***	0.551 ***	0.583 ***
Student-Supervisor Relationship Construction	0.258 ***	0.397 ***	0.319 ***	0.386 ***
Second-order				
Self-Agency	0.496 ***	0.636 ***	0.560 ***	0.655 ***
Academic Agency	0.500 ***	0.592 ***	0.501 ***	0.612 ***
Resource Agency	0.472 ***	0.601 ***	0.564 ***	0.632 ***
Overall	0.541 ***	0.674 ***	0.600 ***	0.700 ***

Note: *** *p* < 0.001.

**Table 6:** Correlations between doctoral student agency and research role identity.

Dimension	Research Role Identity
First-order	
Self-Cognition	0.671 ***
Self-Management	0.471 ***
Academic planning	0.471 ***
Academic autonomy	0.470 ***
Organizational Resource Utilization	0.352 ***
Interpersonal Resource Utilization	0.447 ***
Student-Supervisor Relationship Construction	0.344 ***
Second-order	
Self-Agency	0.633 ***
Academic Agency	0.519 ***
Resource Agency	0.481 ***
Overall	0.592 ***

Note: *** *p* < 0.001.

**Table 7:** Measurement invariance test across grades.

Model	χ^2^	df	χ^2^/df	RMSEA	CFI	TLI	SRMR	ΔRMSEA	ΔCFI	ΔTLI
Configural	1054.218	602	1.751	0.059	0.929	0.918	0.051			
Metric (Weak)	1083.470	622	1.742	0.058	0.928	0.919	0.058	−0.001	−0.001	0.001
Scalar (Strong)	1112.621	642	1.733	0.058	0.926	0.920	0.059	0.000	−0.002	0.001
Strict	1195.557	669	1.787	0.060	0.918	0.914	0.074	0.002	−0.008	−0.006

**Table 8:** Measurement invariance test across discipline categories.

Model	χ^2^	df	χ^2^/df	RMSEA	CFI	TLI	SRMR	ΔRMSEA	ΔCFI	ΔTLI
Configural	1097.362	602	1.823	0.061	0.926	0.914	0.051			
Metric (Weak)	1122.134	622	1.804	0.061	0.925	0.916	0.057	0.000	−0.001	0.002
Scalar (Strong)	1143.539	642	1.781	0.060	0.925	0.918	0.058	−0.001	0.000	0.002
Strict	1190.130	669	1.779	0.060	0.922	0.918	0.062	0.000	−0.003	0.000

## Data Availability

The datasets used in the current study are available from the corresponding author upon reasonable request.

## Appendix A

**Table A1:** Profile of interviewees in higher education research.

No.	Discipline	Academic Qualification	Gender	Identity at the Time of Interview
1	Science	Ph.D.	F	Postdoctoral Researcher
2	Engineering	Ph.D.	M	postdoctoral Researcher
3	Education	Ph.D. candidate	F	Year 4
4	Education	Ph.D. candidate	F	Year 2
5	Science	Ph.D.	F	Year 4
6	Education	Ph.D.	M	Young Faculty
7	Education	Ph.D. candidate	M	Year 2
8	Science	Ph.D. candidate	F	Year 2
9	Management	Ph.D. candidate	F	Year 4
10	Engineering	Ph.D.	M	Postdoctoral Researcher
11	Education	Ph.D.	M	Researcher in Publication
12	Education	Ph.D. candidate	F	Year 4
13	Science	Ph.D. candidate	M	Year 2
14	Science	Ph.D. candidate	F	Year 1
15	Education	Ph.D. candidate	F	Year 4
16	Engineering	Ph.D. candidate	F	Year 5
17	Economics	Ph.D. candidate	M	Year 4
18	Engineering	Ph.D.	M	Young Faculty
19	Philosophy	Ph.D.	M	Young Faculty
20	Science	Ph.D. candidate	M	Year 3
21	Law	Ph.D. candidate	M	Year 3
22	Literature	Ph.D.	M	Postdoctoral Researcher
23	Management	Ph.D.	F	Young Faculty
24	History	Ph.D. candidate	M	Year 4
25	Statistics	Ph.D. candidate	M	Year 3
26	Literature	Ph.D.	F	Postdoctoral Researcher
27	Law	Ph.D. candidate	F	Year 3

## Appendix B. The Initial 43-Item Doctoral Student Agency Scale (DSAS)

A1. I gain a sense of achievement from my doctoral study and research.

A2. Most of the research I conduct during my doctoral study is valuable and meaningful.

A3. During my doctoral studies and research, I believe I can overcome any difficulties I encounter.

A4. I believe I can successfully complete the study and research tasks required for my doctorate.

A5. I believe I can complete my doctoral learning and research tasks effectively.

A6. I can clearly judge my current strengths and weaknesses in doctoral study and research.

A7. I regularly reflect on my shortcomings, summarize experiences, and strive for improvement.

A8. I observe and reflect on the experiences and practices of people around me (e.g., teachers and peers) to gain inspiration.

A9. I manage the balance between my study, research, and other responsibilities (e.g., organizing academic activities, completing tasks assigned by my supervisor).

A10. When encountering emotional stress (e.g., anxiety or tension) during my study and research, I can quickly adjust my state.

A11. I can recognize negative emotions such as stress or anxiety and adjust my state in a timely manner.

A12. When facing tasks or problems in my study and research, I take immediate action to avoid procrastination.

A13. In my study and research, I can quickly put my ideas into practice rather than letting them remain at the conceptual level.

A14. During my doctoral study and research, I can continuously adjust my goals and efforts based on feedback and reflection.

A15. During my doctoral study, I set relatively clear and specific goals for my academic efforts.

A16. During my doctoral study, I am able to break down my goals and carry out academic work systematically according to phased plans.

A17. During my doctoral study, I can stay focused on my established goals and am not easily distracted by other factors.

A18. I usually initiate and carry out my doctoral research independently, rather than passively following others’ arrangements.

A19. In academic communication with my supervisor, I tend to rely on them and lack my own ideas. (r)

A20. I often adjust my communication with my supervisor according to their guidance, to facilitate problem-solving.

A21. I adjust my communication style according to my supervisor’s mentoring approach to facilitate problem-solving.

A22. When I have differing opinions from my supervisor, I actively seek ways to reach consensus.

A23. I consciously build a positive relationship with my supervisor to support my learning and research.

A24. When encountering difficulties in my study and research, I do not avoid them but try my best to think of solutions.

A25. During my doctoral study and research, I can devote sufficient time and energy, maintaining strong perseverance and self-discipline.

A26. I consciously develop good academic habits, such as reading relevant literature regularly, recording ideas, and summarizing viewpoints.

A27. I take the initiative to learn knowledge and skills needed for my study and research.

A28. I consciously practice certain research methods to build my own research expertise.

A29. I systematically conduct research in specific areas to develop my own research field and topic.

A30. I am familiar with my university’s or college’s requirements and regulations regarding progression, evaluation, and graduation.

A31. I actively learn about the requirements and both formal and informal rules related to academic publication, journal selection, and submission.

A32. I am aware of the learning and research resources and opportunities provided by my university and department.

A33. I actively seek opportunities for learning and communication (e.g., lectures, domestic and international conferences) offered by institutional platforms.

A34. I can make full use of the platforms and resources I have to advance my research projects.

A35 I actively build connections with different teachers and peers to expand my academic network.

A36 I actively maintain academic interaction and communication with my supervisor, other faculty members, and peers.

A37. When I encounter difficulties in study and research, I sometimes hesitate to seek help because of shyness, embarrassment, or fear of rejection. (r)

A38. I seek and make use of resources (e.g., networks, projects) from supervisors, peers, and other faculty members both inside and outside the university to support my study and research.

A39. When encountering difficulties in study or research, I know clearly whom to ask for advice or assistance.

A40. In my study and research, I take the initiative to communicate and discuss with supervisors, peers, and others.

A41. My supervisor and peers seldom contact me first, and I often take the initiative to reach out and share information.

A42. In my study and research, I often take the initiative to collaborate with my supervisor, other faculty members, peers, and research group members on academic projects and paper writing.

A43. When encountering problems in my study or research, I proactively look for collaborators to solve them together.

## Appendix C

**Table A2:** The results of the seven common factors extracted through the initial EFA.

Item No.	F1	F2	F3	F4	F5	F6	F7
A38	0.7						
A36	0.69						
A42	0.68						
A43	0.62						
A40	0.59						
A39	0.56						
A34	0.54			0.47			
A12		0.8					
A13		0.74					
A16		0.65					
A17		0.59		0.36			
A25		0.54		0.52			
A15		0.53					
A24		0.45		0.35			
A7			0.67				0.43
A11			0.66				
A8			0.65				0.48
A5			0.65				
A9			0.62				
A6			0.57				
A3			0.56				
A27				0.73			
A26				0.7			
A29				0.69			
A28				0.68			
A19				0.44		0.41	0.43
A22					0.81		
A21					0.75		
A23					0.7		
A18					0.44		
A1						0.77	
A2						0.64	
A4						0.49	
A33	0.47						0.59
A32	0.42						0.58
A31							0.58

## Appendix D. The Final Doctoral Student Agency Scale (DSAS)

### Appendix D.1. Self-Agency

#### Appendix D.1.1. Self-Cognition

A1. I gain a sense of achievement from my doctoral study and research.

A2. Most of the research I conduct during my doctoral study is valuable and meaningful.

A4. I believe I can successfully complete the study and research tasks required for my doctorate.

#### Appendix D.1.2. Self-Management

A11. I can recognize negative emotions such as stress or anxiety and adjust my state in a timely manner.

A7. I regularly reflect on my shortcomings, summarize experiences, and strive for improvement.

A8. I observe and reflect on the experiences and practices of people around me (e.g., teachers and peers) to gain inspiration.

A9. I manage the balance between my study, research, and other responsibilities (e.g., organizing academic activities, completing tasks assigned by my supervisor).

### Appendix D.2. Academic Agency

#### Appendix D.2.1. Academic Autonomy

A27. I take the initiative to learn knowledge and skills needed for my study and research.

A28. I consciously practice certain research methods to build my own research expertise.

A29. I systematically conduct research in specific areas to develop my own research field and topic.

A26. I consciously develop good academic habits, such as reading relevant literature regularly, recording ideas, and summarizing viewpoints.

#### Appendix D.2.2. Academic Planning

A12. When facing tasks or problems in my study and research, I take immediate action to avoid procrastination.

A13. In my study and research, I can quickly put my ideas into practice rather than letting them remain at the conceptual level.

A16. During my doctoral study, I am able to break down my goals and carry out academic work systematically according to phased plans.

A17. During my doctoral study, I can stay focused on my established goals and am not easily distracted by other factors.

A15. During my doctoral study, I set relatively clear and specific goals for my academic efforts.

### Appendix D.3. Resource Agency

#### Appendix D.3.1. Interpersonal Resource Utilization

A35. I actively build connections with different teachers and peers to expand my academic network.

A38. I seek and make use of resources (e.g., networks, projects) from supervisors, peers, and other faculty members both inside and outside the university to support my study and research.

A36. I actively maintain academic interaction and communication with my supervisor, other faculty members, and peers.

A42. In my study and research, I often take the initiative to collaborate with my supervisor, other faculty members, peers, and research group members on academic projects and paper writing.

A43. When encountering problems in my study or research, I proactively look for collaborators to solve them together.

#### Appendix D.3.2. Student–Supervisor Relationship Construction

A22. When I have differing opinions from my supervisor, I actively seek ways to reach consensus.

A21. I adjust my communication style according to my supervisor’s mentoring approach to facilitate problem-solving.

A23. I consciously build a positive relationship with my supervisor to support my learning and research.

#### Appendix D.3.3. Organizational Resource Utilization

A31. I actively learn about the requirements and both formal and informal rules related to academic publication, journal selection, and submission.

A32. I am aware of the learning and research resources and opportunities provided by my university and department.

A33. I actively seek opportunities for learning and communication (e.g., lectures, domestic and international conferences) offered by institutional platforms.
